# Unprovoked Pulmonary Embolism in a Young Patient with Marfan Syndrome

**DOI:** 10.7759/cureus.1655

**Published:** 2017-09-05

**Authors:** Stella Pak, Andrew Kilgore, Rosanne Thornhill, Kyle Rako, Ali Meier, Gavriella Pora, Jillian M Costello, Christine Dee

**Affiliations:** 1 Internal Medicine, Kettering Medical Center; 2 Wright State University Boonshoft School of Medicine; 3 Loma Linda University School of Medicine; 4 Liberty University College of Osteopathic Medicine

**Keywords:** marfan syndrome, pulmonary embolism, chest pain

## Abstract

Marfan syndrome is a rare connective tissue disorder with a prevalence of approximately 2 to 3 per 10,000 individuals. There have been some reports of young patients with Marfan syndrome developing arteriovenous thromboembolism. These events were unprovoked and recurrent. Owing to its rarity, hypercoagulopathy and other metabolic derangement in patients with Marfan syndrome remains largely unknown. Herein, we report a case of a young man with Marfan syndrome who had myocardial infarction and pulmonary embolism. We hope that this case adds to the scant body of knowledge about this patient population.

## Introduction

Marfan syndrome is a connective tissue disorder most often caused by mutations in the extracellular matrix protein fibrillin 1 (FBN1), resulting in decreased elastin content in vessels and increased transforming growth factor-β (TGF-β) activation [1]. The prevalence is approximately two to three per 10,000 individuals. The disorder segregates as autosomal dominant in families with 25% of cases being sporadic, de-novo mutations [1]. The diagnosis of Marfan is primarily clinical and there is no rapid and efficient testing for FBN1 mutations. Characteristic phenotypic findings in the cardiovascular, ocular and skeletal system include aortic root aneurysm, atrioventricular valve thickening, ectopia lentis, long-bone overgrowth, and thoracolumbar scoliosis [1]. Patients with Marfan syndrome frequently suffer from mitral and/or tricuspid prolapse, with possible regurgitation [1]. Ocular complications include retinal detachment and early cataracts or glaucoma [1]. Patients with Marfan syndrome are predisposed to pneumothorax and anterior chest deformities, including pectus carinatum and excavatum, which may lead to a restrictive pattern of lung disease [1]. The most life-threatening manifestation of Marfan syndrome is aortic aneurysm and dissection, which is due to decreased vascular elasticity, and requires lifelong monitoring [1].

Thrombotic events are relatively rare in Marfan patients, indicating a possible protective function for TGF-β activation, potentially through prevention of lipid lesion formation [2]. However, evidence exists of increased risk for hypercoagulability and endothelial dysfunction in Marfan syndrome, both of which may contribute to thromboembolism formation. Bridges, et al. noted increased prothrombotic factor VIII von Willebrand Factor antigen levels in Marfan patients secondary to endothelial injury caused by impaired tensile strength in blood vessels [3]. In the same study, increased thrombomodulin levels, which promotes anticoagulation, were also noted and may serve to balance the hypercoagulability of elevated factor VIII levels [3]. Endothelial injury and dysfunction are known to promote thrombus formation. Wilson, et al. explored the close association between fibrillin and endothelial cells, indicating a possible functional role for fibrillin in the endothelium. The study demonstrated impaired flow-mediated vasodilation, possibly due to dysfunctional mechanotransduction and reduced large-artery distensibility as a result of decreased fibrillin levels [4]. Furthermore, Wilson, et al. described elevated plasma concentrations of endothelial cell products in patients with Marfan syndrome, indirectly indicating endothelial dysfunction [4]. Chung, et al. similarly investigated endothelial dysfunction in patients with Marfan syndrome, demonstrating reduced expression of endothelial nitric oxide synthase (eNOS) and Akt, with decreased cyclic guanosine monophosphate (cGMP) in the aorta [5]. Vascular relaxation through acetylcholine signaling was also diminished, indicating multiple possible mechanisms for impaired flow-mediated vasodilation and increased risk for thrombus formation [5].

Herein, we report a case of a young man with Marfan syndrome and history of myocardial infarction (MI) presented to us with unprovoked pulmonary embolism and second-degree atrioventricular block type 1. There was no precipitating factor for the development of thromboembolism in this young man, with exception of Marfan syndrome.

## Case presentation

A 22-year-old man with Marfan syndrome and a history of unprovoked MI presented to our facility with a sudden onset of left-sided chest pain and worsening dyspnea. His medical history was negative for hypertension, hyperlipidemia, or diabetes mellitus. He described sharp, pinching pain aggravated by deep breathing. There was no history of recent surgery, travel, or immobilization. His vital signs and physical examination were all within normal limits, with exception of marfanoid features, including tall stature, scoliosis, arachnodactyly, and flat feet.

**Figure 1:**
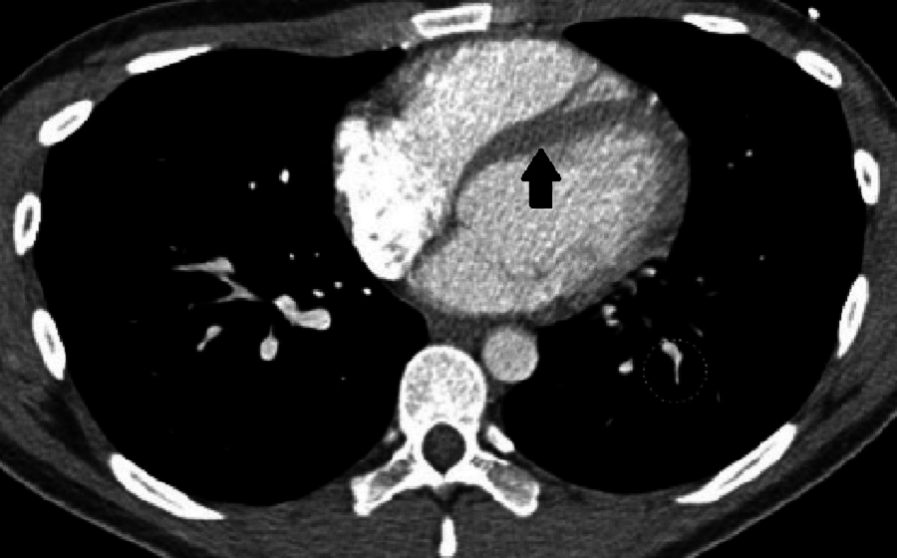
Computerized tomography angiogram of chest. Note the filling defect in left descending pulmonary artery.

Electrocardiogram (ECG) and roentgenogram of chest did not show any abnormalities. Transesophageal echocardiography revealed no evidence of aortic aneurysm or dissection. However, computerized tomography angiogram of chest visualized pulmonary embolism in the left descending pulmonary artery (Figure 1). Our patient was expeditiously started on heparin drip (25,000 units in 250 mL infusion, 0.45 sodium % chloride). His coagulopathy workups, including prothrombin time (PT) (14 seconds) [normal value = 11-15 seconds], partial thromboplastin time (PTT) (26.3 seconds) [normal value = 25-40 seconds], international normalised ratio (INR) (1), factor V Leiden mutation, prothrombin mutation, protein C deficiency, protein S deficiency, antithrombin deficiency, returned negative. He also tested negative for anti-phospholipid antibodies and urine homocysteine. Of note, 24-hour telemetry showed second-degree arteriovenous block type I. On the third day of hospitalization, he reported a remarkable improvement in both chest pain and dyspnea. He was discharged in stable condition on enoxaparin (80 mg) and warfarin (5 mg).

## Discussion

Marfan syndrome has not been proven to be associated with a hypercoagulable state, and very few cases have reported thromboembolic events in Marfan syndrome. However, early diagnosis and management is still critical in these patients due to their already fragile vascular state. Despite negative results from the ECG and roentgenogram, the patient’s history of MI prompted us to perform a thorough workup. Without this history, diagnosis of pulmonary emboli (PE) in this young patient might have been missed.

ECG on our patient revealed an asymptomatic Mobitz Type I second-degree atrioventricular block. Types of arrhythmia prevalent among individuals with Marfan syndrome include atrial fibrillation, atrial flutter, ventricular arrhythmias, bundle branch blocks, and atrioventricular blocks [6-7]. In this population, sudden cardiac death is often attributed to aortic dissection, however, studies have documented sudden cardiac death in patients with known non-sustained ventricular tachycardia despite having non-dissected, non-dilated aortas and normal coronary arteries [8-9]. As the direct correlation between various arrhythmias and serious cardiac complications is further investigated, early recognition and evaluation of arrhythmias in these patients is imperative, allowing such potential complications to be identified and treated, if not prevented.

## Conclusions

Due to the rarity of Marfan syndrome, large scale prospective studies are not feasible, making associations of this disease with rare but lethal complications difficult. By reporting this case study, we hope to contribute to the scant body of knowledge and raise clinical suspicion for thromboembolic events in this group of patients. In doing so, early diagnosis and management can be achieved; thus improving quality of life and survival.
